# *Streptocephalus* diversity in Myanmar, with description of a new species (Branchiopoda, Anostraca)

**DOI:** 10.3897/zookeys.734.21153

**Published:** 2018-02-05

**Authors:** Shu-Sen Shu, D. Christopher Rogers, Xiao-Yong Chen, La-orsri Sanoamuang

**Affiliations:** 1 Southeast Asia Biodiversity Research Institute, Chinese Academy of Sciences, Yezin, Nay Pyi Taw 05282, Myanmar; 2 Kunming Institute of Zoology, Chinese Academy of Sciences, Kunming 650223, China; 3 Applied Taxonomic Research Center, Khon Kaen University, Khon Kaen 40002, Thailand; 4 Kansas Biological Survey, and The Natural History Museum (Biodiversity Institute),; 5 Kansas University, Kansas 66047-3759, USA; 6 International College, Khon Kaen University, Khon Kaen 40002, Thailand

**Keywords:** Diversity, new record, Southeast Asia, *Streptocephalus shinsawbuae* sp. n.

## Abstract

The diversity of anostracans in Myanmar is poorly known. A series of biodiversity surveys had been conducted in Myanmar, and two species of *Streptocephalus* were collected in the central dry zone. *Streptocephalus sirindhornae* Sanoamuang et al., 2000 is reported in Myanmar for the first time, and *Streptocephalus shinsawbuae* **sp. n.** is described as new. *Streptocephalus shinsawbuae* **sp. n.** belongs to the *S. dichotomus* group and is similar to *S. simplex* Bond, 1934 and *S. sahyadriensis* Rogers & Padhye, 2014, but can be distinguished by the form of the male antennal posterior primary ramus and anterior primary ramus apex and egg ornamentation. *Streptocephalus dichotomus* has been reported from Myanmar in the past but was not found in this survey.

## Introduction

The monogeneric Streptocephalidae Daday, 1910 is the largest anostracan family, composed of 65 species (Rogers 2013; Rogers and Padhye 2014), distributed across Africa, Eurasia, Australia, and North America (Daniels et al. 2004). *Streptocephalus* diversity in Asia has been examined in detail, with eleven species were reported from the Middle East to Taiwan (Rogers et al. 2013; Rogers and Padhye 2014, 2015; Shu et al. 2015). Six species are regarded as valid in India (Rogers and Padhye 2014; 2015). Four species have been reported from Southeast Asia, although this region as a whole is poorly studied (Rogers et al. 2013). *Streptocephalus javanus* Brehm, 1955 has been found and described from the island of Java (Vaas 1952; Brehm 1955), *Streptocephalus sirindhornae* and *S. siamensis* have been described from Thailand by Sanoamuang et al. (2000) and Sanoamuang and Saengphan (2006), respectively. Only *Streptocephalus dichotomus* has been reported from Myanmar previously (Belk and Brtek 1995; Sanoamuang et al. 2000).

The Southeast Asia Biodiversity Research Institute (Chinese Academy of Sciences) and the Forest Research Institute, Myanmar, conducted a series of biodiversity surveys in Myanmar from 2015 to 2017. Two *Streptocephalus* species were collected during these efforts: the first records of *S. sirindhornae* Sanoamuang et al., 2000 from Myanmar and a species new to science.

## Materials and methods

Specimens were collected by a hand held dip net and preserved in 95% alcohol in the field. Specimens were examined under a stereo microscope (Zeiss Stemi 508) and a compound microscope (Olympus CX31) in the laboratory. All drawings were made using a camera lucida and images were taken by ToupCam microscope digital camera inside the compound microscope, the egg image (Fig. 4D) was taken at different focal planes and combined automatically by Toupview to increase the depth of focus. The distribution map was produced by ArcGIS based on the GPS information which was collected in the field using a Garmin eTrex 309. Terminology follows Rogers et al. (2013), but to prevent confusion, parallel morphological terminology from Maeda-Martínez et al. (1995) and Sanoamuang et al. (2000) is marked in brackets. All specimens examined were deposited in the Kunming Natural History Museum of Zoology, Kunming Institute of Zoology, Chinese Academy of Sciences.

## Results

### Order: Anostraca Sars, 1867

#### Family: Streptocephalidae Daday, 1910

##### Genus: *Streptocephalus* Baird, 1852

###### Streptocephalus (Streptocephalus) shinsawbuae sp. n.

Taxon classificationAnimaliaAnostracaStreptocephalidae

http://zoobank.org/2E708D52-6149-4C96-92D9-52F08945DB0A

**Figure 1.**
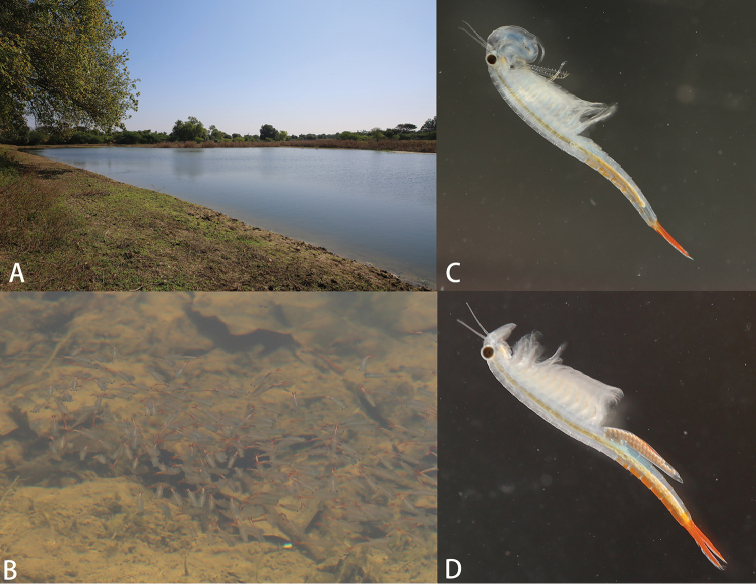
*Streptocephalus shinsawbuae* sp. n. **A** type locality **B** aggregation **C** adult male **D** adult female.

**Figure 2.**
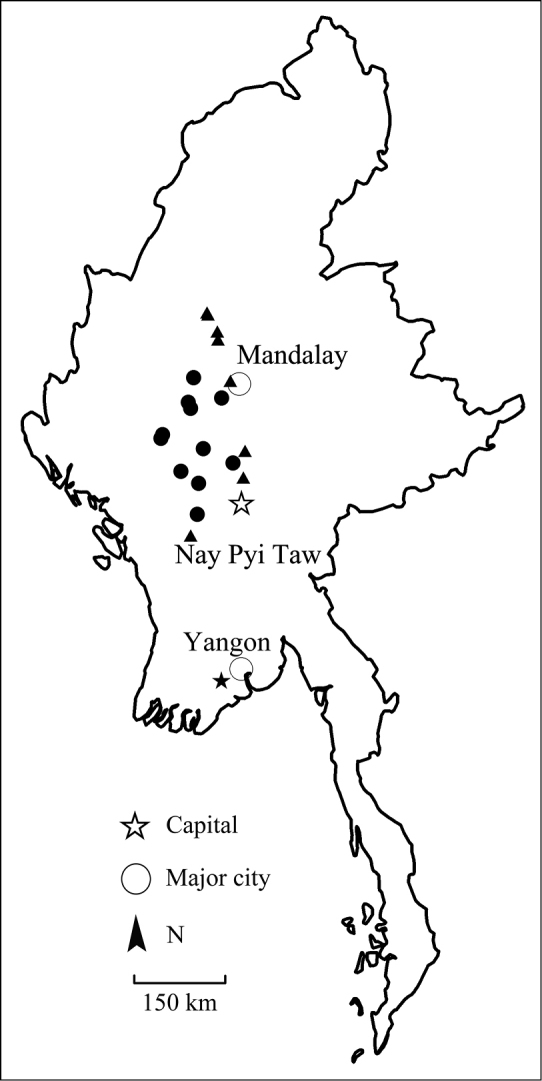
The distribution of *Streptocephalus shinsawbuae* sp. n. (black circles), *S. sirindhornae* (black triangles), and *S. dichotomus* (black star) in Myanmar.

**Figure 3.**
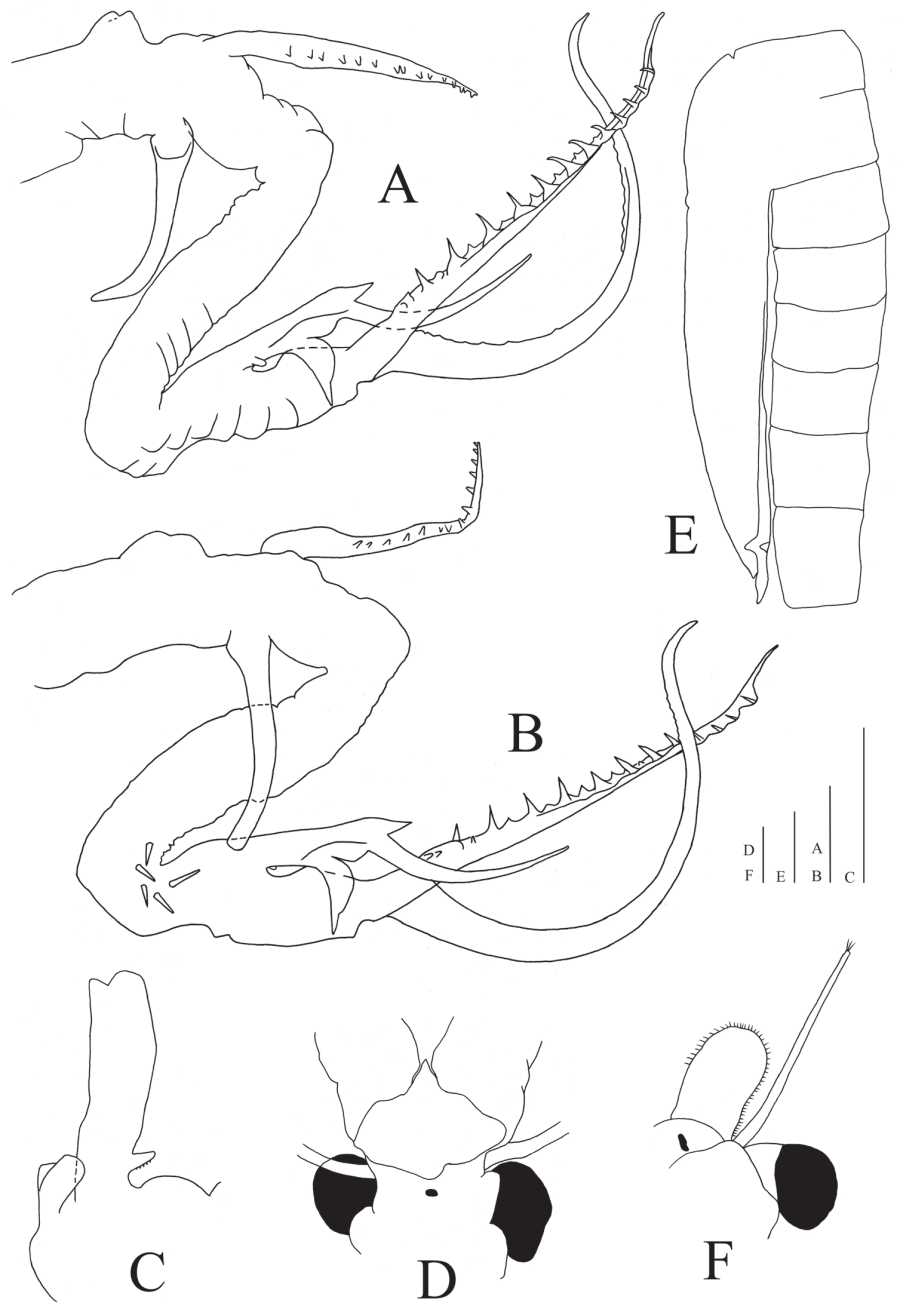
*Streptocephalus shinsawbuae* sp. n. **A** medial view of male second antenna **B** lateral view of male second antenna **C** gonopod, ventral view **D** male head, anterior view **E** brood pouch, left lateral view **F** female head and antennae, right side, anterior view. Scale bars 1 mm.

**Figure 4.**
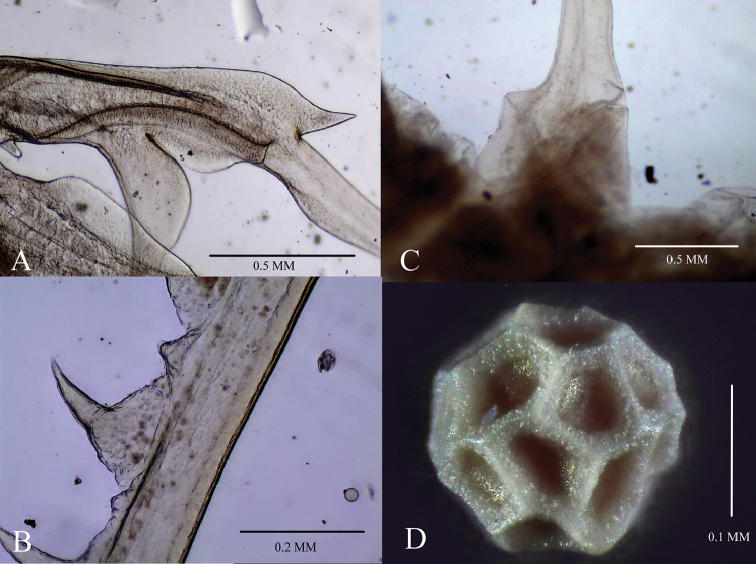
*Streptocephalus shinsawbuae* sp. n. **A** male anterior ramus (thumb) **B** male distal antennomere base **C** male posterior primary ramus (upper finger) primary small spines and hyaline large spines **D** egg.

Figures 1 , 2 , 3 , 4

####### Holotype.

KIZ–CR 2016001, male, collected from type locality on 29 December 2016: SS Shu, XY Chen, T Qin, KM Myint and TS Tin. Type deposited in the Kunming Natural History Museum of Zoology, Kunming Institute of Zoology (KIZ), Chinese Academy of Sciences (CAS).

####### Allotype.

KIZ–CR 2016002, female, same data as holotype.

####### Paratypes.

One male (SEABRI–CR 2016001) and one female (SEABRI–CR 2016002) deposited in Freshwater Biodiversity Laboratory, Southeast Asia Biodiversity Research Institute, Chinese Academy of Sciences, Myanmar, same data as holotype.

####### Type locality.

(Fig. 1A, B) Myanmar: Mandalay Region: Pyawbwe Township: near Yanaung Village: a pond in the southern side of the road from Pyawbwe to No. 1 Highway, 20°33’46.9”N, 95°58’53.70”E, altitude 242 m.

####### Material examined.

**MYANMAR**: Mandalay region: Pyawbwe Township: near Yanaung Village: a pond in the southern side of the road from Pyawbwe to NO. 1 Highway, 20°33'46.9"N, 95°58'53.70"E, altitude 242 m, 25 males and 18 females. Zayetkon Township: a pond near the road from Kyaukpadaung to Nay Pyi Taw, 20°48'51.63"N, 95°26'58.88"E, altitude 430 m, 11 males and 24 females. SAGAING REGION: Monywa and Chaung-U Townships: a pond near Bawditataung Nature Reserve (Laykyun Sekkya Buddha), 22°5'26.47"N, 95°16'30.85"E, altitude 141 m, 6 males and 22 females. Myo Thar Township: a pond near the road from Gway Kone to Myo Thar, 21°43'37.31"N, 95°46'40.34"E, altitude 172 m, 15 males and 8 females. MAGWAY REGION: Yesagyo Township: a pond near the road from Yesagyo to Lingadaw, 21°38'46.22"N, 95°10'56.00"E, altitude 90 m, 10 males and 8 females. Htammakauk Township: a pond near the road from East Kan Dwinn to Ohnbin, 21°4'13.33"N, 94°43'21.61"E, altitude 105 m, 4 males and 3 females. Kyuntaw Township: a pond near the road from Ywathitkyi to Htanpinchaung, 21°0'16.17"N, 94°41'18.05"E, altitude 128 m, 15 males and 22 females. Chaung Kauk Township: a pond near the road from Koebin to Egayit, 19°38'22.09"N, 95°20'25.40"E, altitude 153 m, 6 males and 12 females. Lelu Township: a pond near the road from Taungdwingyi to Magway, 20°11'55.78"N, 95°22'0.62"E, altitude 145 m, 15 males and 13 females. Yenangyaung City, Gyae Gone Township: a pond near the road from Gyae Gone to Wetchok, 20°24'31.38"N, 95°2'57.32"E, altitude 200 m, 5 males and 17 females. All specimens except the type series were collected by SS Shu, XY Chen, T Qin, P Zaw in June and July, 2017, and the locations are marked in Fig. 2.

####### Diagnosis.

*Streptocephalus shinsawbuae* sp. n. is a member of the “*S. dichotomus*” species group, and can be distinguished from its congeners by the following characters: base of second antenna distal antennomere expanded, subquadrate, basal projection absent; antennal appendage with long peduncle, with one (rarely two) fleshy papilla(e), distal geniculations with 5–7 spines; anterior primary ramus with a digitiform basoposterior spine, ending distally as a triangular, lamellar projection, anterior ramus posterior branch with a subdistal and shallow notch; posterior ramus biramous, posteriolateral branch with two groups of crenulations, posterior primary ramus with two longitudinal rows of spines, distal tenth slightly curved anteriorly; egg with large, basically pentagonal polygons, separated by vertical ridges.

####### Description.

***Male.*** (Fig. 1C) *Body* length (from anterior margin of head to posterior margin of telson, not including cercopods) from 14.5 mm to 20.5 mm, average 17.7 mm.

*Head* round, subcylindrical. Cephalic appendage (Fig. 3D) short, triangular, unbranched, length ~30 % of second antenna proximal antennomere. First antenna filiform, extending beyond second antenna distal antennomere base, apex blunt, bearing three subequal long setae and two short setae. Second antenna (Fig. 3B) extending posteriorly to eighth thoracic segment. Proximal antennomere subcylindrical, length nearly four times width, medial surface smooth, without setae or pulvinus. Distal antennomere 0.9 times as long as proximal antennomere, laterally directed, smooth, curving medially in distal half; apex expanded and rounded to truncate; base expanded, subquadrate (Fig. 4B), basal projection absent.

Antennal appendage (Fig. 3A) with long peduncle (*sensu* Maeda-Martinez et al. 1995), length 1.6 times second antenna proximal antennomere, subcylindrical, without pulvinus. Peduncle anteromedial surface proximally with one fleshy papilla, half as long as peduncle, bearing a ventrolateral, longitudinal row of 12-14 subequal spines. Length of middle peduncle pseudosegment (between geniculations) more than half peduncle. Antennal appendage peduncle distal geniculations with a lateral longitudinal row of 5–7 spines; spines smooth, acute, with length approx. half of peduncle width. Antennal appendage apical cheliform structure (“hand” in Maeda-Martínez et al. 1995) strongly developed, with anterior ramus (“thumb”) and posterior ramus (“finger”).

Anterior primary ramus (Figure 4A) (the so called “thumb” see Maeda-Martínez et al. 1995) with an anterior, longitudinal, chitinized carina, ending distally as a triangular, lamellar projection, directed distally. Anterior ramus (“thumb”) posterior branch (“spur”) smooth, arcuate, recurving posteriorly approx. 80°, with a subdistal, ventral, shallow notch. “Spur” distoventral margin broadly curving into triangular gap. Triangular gap, becoming a narrow, deep cleft between “spur” and anterior ramus (“thumb”), at least as long as opening width. Anterior ramus (“thumb”) gently arcuate, curving anteriorly approximately 30°, apex acuminate.

Posterior ramus (“finger”) biramous and longer than anterior ramus. Posterior margin in lateral view near rami confluence with a shallow emargination. Posteriolateral branch (“lower finger” in Sanoamuang et al. 2000) arcuate, broadly curved to ~160°, with apex bent nearly 90° distally, nearly attaining primary ramus (“upper finger”) apex. Posteriolateral branch anterior margin subcrenulate in basal third, crenulate proximally in distal third. Posterior primary ramus (“upper finger”) straight, directed distally, subequal in length to peduncle, with distal tenth slightly curved anteriorly. Anterior surface with two longitudinal rows of spines (Fig. 4C). The lateral spine row bears small, wide based spines, from branch confluence to three fourths the length of the ramus. The medial row bears hyaline spines in a series of medial hyaline lamellae, larger than the lateral spines, with tumid bases and aciculate apices. Medial spines increase in size gradually along proximal three fourths, and become more slender and arcuate in distal fourth. Most lamellae developed and connected, with apical half rotating to the medial side of the branch. The medial spine row distal apex ends subdistally on branch.

Labrum large, triangular, middle compress, apex directed posteriorly. Mandible, first and second maxillae as typical for the genus.

Eleven pairs *thoracopods*, increasing in size from the first pair to the fifth pair, then decreasing posteriorly. The structures of praeepipodites and epipodites typical for genus. Fifth thoracopod endite I and II with closely set, long plumose marginal setae. Endite I submargin with three widely spaced spines, the distal two are single, the proximal one with a basal spinule. Endite II submargin with two closely spaced spines, distal spine short, proximal spine long. Endite III–V with 3, 2, 2 long plumose setae and 2, 2, 1 spine(s), respectively, with small setae in proximal half. Endopodite broad, margin distal half with sparse plumose setae, each with 1–6 basal spinulae. Exopodite linguiform, margins with closely set plumose setae, longest distally, most setae with basal spinule. Epipod oval, without setae and spines, prae-epipod broadly oval, margins with small hooks.

*Genital segments* smooth, with lateral linguiform outgrowths. Gonopod (Fig. 3C) cylindrical, with a basomedial spiniform outgrowth, bearing four denticles medially. Everted gonopod elongate, distal end expanded, extending to the distal margin of abdominal segment IV, with a lateral, longitudinal row of spines from base to apex.

*Abdomen* and *cercopods* as typical for the genus.

***Female.*** Body smaller than male, body length from 14.0 to 17.5 mm, average 15.4 mm (Fig. 1D).

First antennae 2.2 times length of eye plus peduncle and 1.6 times length of second antennae, apex blunt, with three subequal long setae. Second antennae (Fig. 3F) broad, oval, smooth, apex round, margins bearing short sparse setae. Thorax smooth. Thoracopods as in male.

Brood pouch (Fig. 3E) elongate, fusiform, extending to the middle or apex of abdominal segment V in most specimens, less frequently extending to segment IV or segment VI.

Egg (Fig. 4D) subspherical, approx. 200 μm in diameter, with large, basically pentagonal polygons, separated by vertical ridges, polygons approx. 40 μm in diameter, and with broad floors.

####### Etymology.

The specific epithet *shinsawbuae* refers to Queen Shin Sawbu (1453–1460) who facilitated more than 50 years of peace in Myanmar.

####### Ecology.

During the sampling at the type location in June, 2017, the pond had a water temperature of 37.6 °C, a pH of 8.3, conductivity of 117μS/cm, and the dissolved oxygen was 5.9 mg/L. One species of clam shrimp, *Cyzicus pilosus* Rogers, Thaimuangphol, Saengphan, and Sanoamuang, 2013 was also collected.

####### Remarks.

*Streptocephalus shinsawbuae* sp. n. is a member of the “*S. dichotomus*” species group, which includes *S. dichotomus* Baird, 1860, *S. echinus* Bond, 1934, *S. longimanus* Bond, 1934, *S. sahyadriensis* Rogers & Padhye, 2014, *S. simplex* Gurney, 1906, and *S. sirindhornae* Sanoamuang et al., 2000. This group is separated from all other *Streptocephalus* in that the posterior ramus (finger) is biramal. Of the six species in this group, *S. shinsawbuae* sp. n. is readily separated from other congers by the single papilla on the antennal appendage peduncle. Of approximately 120 male specimens of *S. shinsawbuae* sp. n., only one male from Magway (20°11'55.78"N, 95°22'0.62"E) had two papillae. This papilla in all other species of group is absent, or numbers three or more.

*Streptocephalus shinsawbuae* sp. n. is most similar to *S. sahyadriensis*. Both species have two longitudinal rows of spines on the antennal appendage posterior ramus (finger), and the anterior primary ramus (thumb) bears a small basoposterior spine. However, they can be separated by: (1) the shape of the posterior primary ramus (upper finger), which is straight in the proximal nine tenths, with the apex arcing anteriorly in *S. shinsawbuae* sp. n. vs. arcing distolaterally in the distal third 90° in *S. sahyadriensis*; (2) the posterior ramus posteriolateral branch (lower finger) has two groups of crenulations along the anterior margin in *S. shinsawbuae* sp. n. vs. only one group subdistally in *S. sahyadriensis*; (3) the anterior primary ramus apex shoulder is triangularly acute in *S. shinsawbuae* sp. n. vs. rounded in *S. sahyadriensis*; (4) the anterior primary ramus (thumb) basoposterior spine is digitiform in *S. shinsawbuae* sp. n. vs. triangular in *S. sahyadriensis*.

*Streptocephalus shinsawbuae* sp. n. is similar to *S. simplex* in having unbranched posterior primary ramus (upper finger), and acute anterior primary ramus (shoulder) apex, but they can be separated by: (1) the posterior ramus posteriolateral branch (lower finger) having two crenulated areas along anterior margin in *S. shinsawbuae* sp. n. vs. smooth in *S. simplex*; (2) the anterior primary ramus (shoulder) apex is triangular in *S. shinsawbuae* sp. n. vs. parallel sided in S. *simplex*; (3) anterior primary ramus (thumb) bearing a basal digitiform spine in *S. shinsawbuae* sp. n. vs. absent in *S. simplex*.

The eggs of *S. shinsawbuae* sp. n. have pentagonal polygons, which are very similar to those of both *S. echinus* and *S. longimanus*. From *S. simplex* it can be readily distinguished by the triangle polygons. In addition, the egg ridges are broad and deep in *S. shinsawbuae* sp. n. vs. narrow and shallow in S. *sahyadriensis*.

###### Streptocephalus (Streptocephalus) sirindhornae

Taxon classificationAnimaliaAnostracaStreptocephalidae

Sanoamuang, Murugan, Weekers, & Dumont, 2000

####### Remarks.

*Streptocephalus sirindhornae* is the most widely distributed member of the genus in Southeast Asia, with previous records from: Thailand, Laos, Cambodia, and China (Sanoamuang et al. 2000; Rogers et al. 2013; Shu et al. 2015). Some characters vary in different populations (Shu et al. 2013), and the materials collected from Myanmar is more similar to the Thai populations, with a deep depression on the posterior ramus ventral margin and unequal apical subrami on the posterior ramus.

Our materials were collected from the central dry zone of Myanmar including Mandalay, Magway, and Sagaing regions (Fig. 2). These are the most western records for this species. The distributional range of *S. sirindhornae* in Myanmar overlaps with that of *S. shinsawbuae* sp. n. (Fig. 2); however, the two species were not found co-occurring in the same pool.

####### Material examined.

MYANMAR: MANDALAY REGION: Nyaung Lunt Township: rice field near the road from Nyaung Lunt to Yamethin, 20°18'9.26"N, 96°9'51.08"E, altitude 189 m, 12 males and 22 females. Hlaingdet Township: a pond near the road from Meiktila to Yin Mar Bin, 20°46'53.80"N, 96°11'30.24"E, altitude 162 m, 8 males and 15 females. SAGAING REGION: Kanbalu Township: Kaing Taw Village: rice field near the road from Kanbalu to Chatthin Wildlife Sanctuary, 23°15'22.53"N, 95°30'54.45"E, altitude 169 m, 5 males and 3 females. Kanbulu Township, Bugon Township and Ywa Zin Township: a pond near the road from Kanbulu to Shwebo, respectively, 23°13'21.22"N, 95°31'55.57"E, altitude 181 m, 3 males and 5 females; 22°55'1.54"N, 95°41'56.33"E, altitude 160 m, 9 males and 15 females; 22°46'30.75"N, 95°42'39.15"E, altitude 152 m, 14 males and 8 females. Saye Township: rice field near Saye Lake, 22°2'14.29"N, 95°55'46.90"E, altitude 86 m, 14 males and 11 females. MAGWAY REGION: Aunglan Township: a pond near the road from Pyay (Prome) to Taungdwinggyi. All specimens were collected by S.S. Shu, X.Y. Chen, T. Qin, P. Zaw in June and July, 2017, and the locations are marked in Fig. 2.

###### Streptocephalus (Streptocephalus) dichotomus

Taxon classificationAnimaliaAnostracaStreptocephalidae

Baird, 1860 (sensu Velu and Munuswamy 2005)

= Branchipus bengalensis Alcock, 1896, *fide* Gurney, 1906.

####### Remarks.

*Streptocephalus dichotomus* is widely distributed in the Indian subcontinent, Sri Lanka, and Pakistan (Selvarajah and Costa 1979; Belk and Esparza 1995; Velu and Munuswamy 2005; Rogers and Padhye 2014, 2015). Belk and Brtek (1995) reported this species from Yangon, Myanmar (Burma), and later, it was regarded as introduced (Sanoamuang et al. 2000). This species was not collected during our surveys.

## Discussion

*Streptocephalus shinsawbuae* sp. n. is the seventh species described from the *S. dichotomus* species group. The number of antennal peduncle papillae was used to separate some Indian species of *Streptocephalus* (Belk and Esparza 1995). Velu and Munuswamy (2005) suggested that this character is important in *Streptocephalus* taxonomy. All of our material bears a single papilla, except one specimen from Magway which has two, the larger papilla as described, with the smaller thin, short, and bare. This one aberrant specimen aside, we think that the single peduncle papilla character is stable, and this character readily allows it to be distinguished from all subgeneric species. This arrangement of papillae appears to fill a gap in the *S. dichotomus* group where the number of papillae is either none (*S. echinus*) or three or more.

Rogers and Padhye (2014) provided the keys for species of Asian *Streptocephalus* species. *Streptocephalus shinsawbuae* sp. n. would key out to couplet 8, which terminates with *S. simplex* and *S. longimanus*. We propose the following amendment to that key at couplet 6 to accommodate the new species:

**Table:** 

6(5)	Antennal peduncle papillae three or more; antennal appendage posterior ramus (“finger”) with lateral sickle shaped lateral subramus inerm on proximal half, crenulate on distal half	**7**
6’	Antennal peduncle papillae absent; antennal appendage posterior ramus with lateral sickle shaped lateral subramus with a longitudinal row of spines, at least proximally with crenulations (immatures); India	***Streptocephalus echinus* Bond, 1934**
6’’	Antennal peduncle papillae one (rarely two); antennal appendage posterior ramus (“finger”) with lateral sickle shaped lateral subramus inerm on proximal half, crenulate on distal half; Myanmar	***Streptocephalus shinsawbuae* sp. n.**

Rogers et al. (2013) predicted that six species of *Streptocephalus* may occur in Southeast Asia. Our survey found two *Streptocephalus* species in Myanmar, bringing the total known number for this nation to three. The diversity of large branchiopods is poorly known in most of Southeast Asia, but it is probably fairly rich, especially since five species (Rogers et al. 2016a, b) have been reported or described since the last comprehensive review of the region (Rogers et al. 2013).

## Supplementary Material

XML Treatment for Streptocephalus (Streptocephalus) shinsawbuae

XML Treatment for Streptocephalus (Streptocephalus) sirindhornae

XML Treatment for Streptocephalus (Streptocephalus) dichotomus
